# Comparative assessment of phenolic composition profile and biological activities of green extract and conventional extracts of *Salvia sclarea*

**DOI:** 10.1038/s41598-024-51661-z

**Published:** 2024-01-22

**Authors:** Mohammed Mansour Quradha, Mehmet Emin Duru, Selcuk Kucukaydin, Alfred Ngenge Tamfu, Mudassar Iqbal, Hamida Bibi, Rasool Khan, Ozgur Ceylan

**Affiliations:** 1 College of Education, Seiyun University, Seiyun, Yemen; 2 Department of Chemistry, Faculty of Science, Mugla Sitki Kocman University, Mugla, 48000 Turkey; 3 Department of Medical Services and Techniques, Koycegiz Vocational School of Health Services, Mugla Sıtkı Kocman University, Koycegiz/Mugla, Turkey; 4 Department of Chemical Engineering, School of Chemical Engineering and Mineral Industries, University of Ngaoundere, 454 Ngaoundere, Cameroon; 5 Department of Agricultural Chemistry and Biochemistry, The University of Agriculture, Peshawar, 25000 Pakistan; 6 Department of Environmental Sciences, Abdul Wali Khan University, Mardan, Pakistan; 7 Institute of Chemical Sciences, University of Peshawar, Peshawar, 25120 Pakistan; 8 Food Quality Control and Analysis Program, Ula Ali Kocman Vocational School, Mugla Sitki Kocman University, Ula Mugla, 48147 Turkey; 9 Pharmacy Department, Medical Sciences, Aljanad University for Science and Technology, Taiz, Yemen

**Keywords:** Enzymes, Drug screening

## Abstract

In recent years, there have been an attempt to develop safe and environmental friendly solvents to replace conventional solvents, and use for extraction bioactive compounds from natural sources. A current investigation involved the preparation of green, methanolic, and ultrasonic extracts of *S. sclarea*, and compared their phenolic profiling using HPLC–DAD, antibacterial, antioxidant, and enzyme inhibition activities. The HPLC–DAD analysis revealed that Rosmarinic acid was the main content in all extracts, with Ellagic acid only present in the green extract. The green extract exhibited superior anti-biofilm activity against *S. Aureus* and *E. Faecalis* compared to the other extracts at MIC concentration. Furthermore, the green extract also displayed the highest inhibition of swarming motility in *P. Aeruginosa* with inhibition range 68.0 ± 2.1 (MIC) to 19.5 ± 0.6 (MIC/4). and better enzyme inhibitory activity against BChE (with IC_50_ = 131.6 ± 0.98 µg/mL) and AChE (with inhibition 47.00 ± 1.50%) compared to the other extracts; while, the ultrasonic extract showed strong inhibition of violacein production by *C. Violaceum* with a inhibition range 05.5 ± 0.1 (MIC/32) to 100 ± 0.00 (MIC), followed by the green extract with a inhibition range 15.0 ± 0.5 (MIC/8) to 100 ± 0.00 (MIC), additionally, the ultrasonic and methanoic extracts showed significant activity against urease enzyme with (IC_50_ = 171.6 ± 0.95 µg/mL and IC_5 0_ = 187.5 ± 1.32 µg/mL) respectively. Both the green and methanolic extracts showed considerable antioxidant activities, as *β*-carotene-linoleic acid (IC_50_ = 5.61 ± 0.47 µg/mL and 5.37 ± 0.27 µg/mL), DPPH^**·**^ (IC_50_ = 19.20 ± 0.70 µg/mL and 16.31 ± 0.23 µg/mL), ABTS^**·**+^(IC_50_ = 8.64 ± 0.63 µg/mL and 6.50 ± 0.45 µg/mL) and CUPRAC (A_0.5_ = 17.22 ± 0.36 µg/mL and 12.28 ± 0.12 µg/mL) respectively, likewise the green extract performing better in metal chelating compared to the other extracts. The green extraction is reported as a cost effective and solvent free method for extracting natural products that produces compounds free of toxic chemicals. This could be the method to be used in the industries as a renewable method.

## Introduction

For centuries, natural products in traditional medicine, have been considered as an important source of treatment for many diseases. According to the World Health Organization (WHO) reports, over 80% of the world's population has used traditional medicines as part of their primary health care^1,2^. Natural products and their derivatives have been an important source of new biologically active compounds^3^. In addition, the therapeutic properties of bioactive compounds obtained from natural sources are affected by geographical location for the same species^4^. Historically the plant materials have been used to the treat various illnesses. The bioactive compounds isolated from natural sources have become a lead source of synthetic drugs^5,6^. When isolated from their natural source, these bioactive metabolites are primarily found in low concentrations^7^. Similarly, conventional extraction procedures require more resources. Therefore, new methods for extracting bioactive compounds are an attractive option for obtaining significant quantities from natural sources. The extraction step is considered one of the most important steps in preparing samples for the phytochemical investigation. The conventional solvents used in the extraction process are non-renewable petroleum-based volatile organic compounds. These solvents can possess a severe threat to the environment and human health^8^. Green technology plays an important role in replacing common petrochemical solvents with inherent toxicity and high volatility, leading to the evaporation of volatile organic compounds into the atmosphere^9^. Recent studies have revealed; that natural solvents are more efficient than petroleum organic solvents in extracting plants metabolites and food products^9,10^. Moreover, they are easy to prepare and have a better cost-to-benefit ratio. Green extraction brings forth a multitude of positive features. These include, but are not limited to: (1) reducing cost and mitigating risk associated with the utilization of organic solvents. (2) Facilitating scalability and expansion of the extraction process. (3) Enhancing safety measures by minimizing the risk of overpressure and explosions^8^. *Salvia sclarea* is a medicinal plant that classified under the genus Salvia, belonging to the Lamiaceae family. It possesses aromatic properties and is naturally found in the North Africa, Northern Mediterranean and Centre Asia^11,12^, it has been extensively studied and has demonstrated various beneficial properties including anti-inflammatory, antioxidant, antitumor, antidiabetic, sedative, and antibacterial effects^13,14^. Additionally, the essential oil extracted from *S. sclarea* is utilized for its antiseptic, antidepressant, carminative, aphrodisiac, and antispasmodic properties^15^. This study aims for the first time to evaluate and compare the ability of green extract and conventional extracts of *S. sclarea* through HPLC profile. The extracts were also studied for their antibacterial activities including biofilm formation inhibition, violacein production, as well as swarming motilities and enzyme inhibition activities including anti-cholinesterase, anti-urease and antioxidant activities.

## Materials and methods

### Collection of plant

The *Salvia sclarea* was collected after getting permission from departmental ethical committee following the protocol described by the Ernst 1995^16^. Aerial parts of *S. sclarea* were collected randomly from Mugla-Mentese, on yerkesik road, altitude: 60 m. The taxonomic identification of this plant was determined by Dr. Hasan Ylldlnm from Ege University in Izmir, Turkey, conducted the taxonomic identification of this plant. The voucher specimen was then stored at the Natural Products Laboratory, Faculty of Science, Mugla Sltkl Koçman University, with the voucher number TSP1012. The collected sample was dried in the shade, ground, and subsequently used for extraction.

### Extraction procedures

#### Methanol extraction

Exact (10 g) of *S. sclarea* (aerial parts) were soaked in 300 mL of methanol at room temperature for 72 h. The mixture was then filtered through a cellulose filter paper with a pore size of 0.45 μm. The methanol was removed by evaporation under reduced pressure using a rotary evaporator (Heidolph, Hei-Vap Value Gl, Germany), resulting in the formation of a dry extract with a brownish-green gummy consistency.

#### Ultrasonic assisted extraction

Ultrasonic cleaner, [model lab. Ult.4030) comprised of a processor unit Q600 (500 W, 40 kHz)] was used for extraction. (10 g) of *S. sclarea* (aerial part) were transferred into a 500 mL conical beaker, 300 mL of methanol was added, the beaker was sealed, and the extraction was performed for 25 min. After extraction, the solution was filtered by using a filter paper (0.45 μm cellulose) and the extract was concentrated by using rotary evaporator.

#### Green extraction

##### Preparation of natural deep eutectic solvents (NADES)

Green extraction of *S. sclarea* was carried out using natural deep eutectic solvent (NADES) (citric acid: glucose) was prepared following the protocol described previously^17,18^. Briefly, citric acid and glucose were mixed at molar ratio (1:2) and located into sealed conical flask 250 mL; the mixture was subjected to constant string and heated to a temperature of 80 °C until homogeneous liquid was achieved.

##### Crude extraction using green solvent

Extraction was performed by adding the green solvent to 3 g of plant material in a sealed conical flask, the; distilled water (20 mL) was also added to lower the viscosity. The mixture was heated to a temperature of 40 °C and stirred for a duration of 10 h. Afterward, the sample was transferred into a falcon tube with a capacity of 50 mL. The falcon tube was then subjected to centrifugation at a speed of 4000 for a period of 20 min. The resulting sediments were discarded, and the remaining liquid portion (supernatant) was filtered using a cellulose paper with a pore size of 0.45 μm. Following filtration, the aqueous mixture underwent evaporation using a rotary evaporator, followed by freeze-drying. Finally, the freeze-dried product was stored at a temperature of 30 °C for additional investigation.

### Determination of phenolic composition

We used a Shimadzu 20 AT series high-performance liquid chromatograph with a diode array detector (HPLC–DAD) from Shimadzu Cooperation in Kyoto, Japan to analyze the phenolic compounds. The extracts were dissolved in a mixture of methanol and water (20:80) and filtered through a 0.2 μm disposable LC filter disk to remove any suspended particles before loading onto an Intersil ODS-3 reverse phase C18 column for distinction and detection^19^. The mobile phase system consisted of 0.5% acetic acid in water as mobile phase A, while; 0.5% acetic acid in methanol as mobile phase B. The elution gradient was as follows: 0–10% B (0–0.01 min); 10% B (0.01–5 min); 20–30% B (5–15 min); 30% B (15–25 min); 50–65% B (25–30 min); 65–75% B (30–40 min); 75–90% B (40–50 min); and finally, back to 10% B (50–55 min). The detection was carried out at a wavelength of 280 nm. To characterize the phenolic compounds, we compared their UV data and retention times with commercial standards. For quantification, we established a calibration curve by injecting known concentrations (0.0, 0.00782, 0.01563, 0.03125, 0.0625, 0.125, 0.25, 0.5, and 1.0 ppm) of standard compounds. In total, we used 26 standard phenolic compounds including Gallic, Protocatechuic, P-coumaric, P-hydroxy benzoic, Chlorogenic, Caffeic, 3-Hydroxy benzoic, Syringic, Ellagic, Eerulic, Rosmarinic, Trans-cinnamic acids, Catechin, Pyrocatechol, 6,7-Dihydroxy coumarin, Vanillin, Taxifolin, Coumarin, Rutin, Myricetin, Luteolin, Quercetin, Hesperetin, Apigenin, Kaempferol, and Chrysin. The results were reported in (μg/g) of dry weight.

### Biological activities

#### Antimicrobial activity

The study employed a range of microorganisms, including *Staphylococcus aureus* ATCC 25923, *Enterococcus faecalis* ATCC 29212, *Escherichia coli* ATCC 25922, Candida albicans ATCC 10239, *Chromobacterium violaceum* CV 12472, *Chromobacterium violaceum* CV026, and *Pseudomonas aeruginosa* PA0 1.

#### Determination of minimum inhibitory concentrations

For determination of the MICs, a microliter broth dilution method recommended by Clinical Laboratory Standards Institute was followed^20^. The test medium used was Mueller–Hinton broth and the bacterial density was 5 × 10^5^ colony-forming units (CFU)/mL. The MIC was defined as the lowest compound or extract concentration that showed no visible growth. Inoculation of cell suspensions (100 μL) was done on 96-well microliter plates containing either extract(s) or varied concentrations (1, 0.5, 0.25, 0.125, 0.0625, 0.0312 mg/mL). The microplates were incubated at 37 °C and read after 24 h.

#### Effect of extract on bacterial biofilm formation

To examine the effect of *S. sclarea* extract(s) on bacterial biofilm formation, a microplate biofilm assay was conducted using a range of extract concentrations, including 1, 1/2, 1/4, and 1/8 MIC^21^. Briefly, overnight cultures of the test microorganisms were diluted to 1% and added to 200 μL of fresh Tryptose-Soy Broth (TSB) supplemented with 0.25% glucose, and then incubated in the absence and presence of extract(s) without agitation for 48 h at 37 °C. Wells containing only TSB and cells were used as controls. After incubation, the wells were washed with water to eliminate planktonic bacteria, and the remaining bacteria were stained with a 0.1% crystal violet solution for 10 min at room temperature. The wells were washed again to eliminate excess crystal violet, and 200 μL of 33% glacial acetic acid in ethanol was added to each well. The resulting solution was shaken and pipetted, and then transferred (125 μL) to a sterile tube. The volume was adjusted to 1 mL with distilled water. Finally, optical density (OD) of each well was measured at wavelength of 550 nm using a Thermo Scientific Multiskan FC instrument (Vantaa, Finland). The percentage of inhibition caused by the tested extracts was calculated using the following formula:

$$\mathrm{Biofilm\, inhibition }(\mathrm{\%}) = \frac{{OD550}_{Control}-{OD550}_{Sample}}{{OD550}_{Control}}\times 100$$

#### Bioassay for quorum-sensing inhibition (QSI) activity using *C. violacium* CV026

In order to assess the quorum-sensing inhibition (QSI) activity of *S. sclarea* extract(s), a bioassay using *C. violacium* CV026 was conducted with some modifications as described elsewhere^22^ with small adjustments. Five milliliters of warm molten Soft Top Agar (consisting of 2.0 g tryptone, 1.3 g agar, 200 mL deionized water and 1.0 g sodium chloride) was seeded with 100 μL of overnight *C. violacium* CV026 culture, followed by the addition of 20 μL of 100 μg/mL C_6_HSL as an exogenous AHL source. The mixture was gently mixed and immediately poured over the surface of a solidified LBA plate as an overlay. Wells with a diameter of 5 mm were made on each plate after the overlay had solidified. All well was then occupied with 50 μL of sub-MIC concentrations of filter-sterilized extract. The development of a white or cream-colored halo around the well against a purple lawn of activated *C. violacium* CV026 bacteria indicated QSI, the antimicrobial activity of the extracts was determined by observing the clear halo around the samples. To determine the limit of detection, serial dilutions of the extracts were made using LB broth as a diluent, ranging from (1:1 to 1:8). The end points were determined as the lowest dilution of extract that showed noticeable inhibition of violacein synthesis. Each experiment was repeated three times and the assay plates were incubated at 30 °C for 3 days. After incubation, the diameters of the quorum sensing inhibition zones were measured.

#### Violacein inhibition assay using *C. violacium* CV12472

Qualitative analysis was conducted on extracts of *S. sclarea* to determine their potential as quorum sensing inhibitors (QSI) against *C. violaceum* ATCC 12472^23^. An overnight culture of *C. violaceum* (adjusted to 0.4 OD) was added to sterile microliter plates containing 200 μL of LB broth. The plates were then incubated in the presence and absence of sub-MICs of the extract. A positive control was included using LB broth containing *C. violaceum* ATCC 12472. The plates were incubated at 30 °C for 24 h and observed for a decrease in violacein pigment production, with absorbance readings taken at 585 nm. The percentage of violacein inhibition was calculated using the following formula:

$$\mathrm{Violacein \, inhibition }(\mathrm{\%}) = \frac{OD\, 585 \, control- OD\, 585 \, sample }{OD \, 585 \, control}\times 100$$

#### Swarming motility inhibition on *P. aeruginosa* PA01

The inhibition of swarming motility assay by *S. sclarea* extract(s) was conducted according to a previously described method^24^. In summary, *P. aeruginosa* PAOI strain overnight cultures were point inoculated at the centre of swarming plates containing of 0.5% NaCl, 1% peptone, 0.5% filter-sterilized d-glucose and 0.5% agar. Different concentrations of extract (50, 75, and 100 μg/mL) were added to the plates, while a plate without extract served as the control. The plates were then incubated upright at an appropriate temperature for 18 h. The migration of swarming was observed by tracking the movement of bacterial cells at the swarm fronts.

#### Anticholinesterase activity

The extracts of *S. sclarea* was tested for its anticholinesterase activity by determining its inhibitory effects on acetylcholinesterase and butyrylcholinesterase using a spectrophotometer and following the protocol described by Ellman with slight modifications^25^. The reference compound used was Galantamine, and the IC_50_ values were calculated using a program derived from the anticholinesterase graph. The inhibitory activity was expressed as percentages (% inhibition) relative to sample concentrations (μg/mL).

#### Anti‑urease activity

To evaluate the inhibitory activity of urease enzyme by each extract, ammonia production was determined using the indophenol method with a microplate reader^26^. In brief, 25 μL of enzymatic urease solution obtained from jack bean source, 50 μL of 100 mM Urea, and 100 mM Sodium phosphate buffer (pH 8.2) were mixed and incubated at 30 °C for 15 min after adding the sample (10 μL extracts). Then, 45 μL of Phenol reagent and 70 μL of Alkali reagent were added to each well. After 50 min of incubation, the absorbance was recorded at wavelength of 630 nm using a microplate reader. Thiourea was used as the reference compound, and the results were expressed as a 50% inhibitory concentration (IC_50_).

#### Antioxidant activities

Antioxidant properties of all extracts were assessed using various methods, which included: (1) *β*-carotene-linoleic acid assay, (2) DPPH assay, (3) ABTS assay, (4) CUPRAC assay and (5) metal chelating assay. Inhibition of lipid peroxidation activity was performed by using *β*-carotene-linoleic acid test system according to the standards protocol developed^27^ with minor modifications^28^. The DPPH assay was performed by using a spectrophotometer according to typical methods documented previously^29,30^. The ABTS^+^ assay was carried out as described previously by Marco, 1968^31^ with slight modification^32^. The cupric reducing antioxidant capacity (CUPRAC) was evaluated by following the method published previously^33^. α-Tocopherol and butylated hydroxyanisole (BHA) were used as antioxidant standards to compare the DPPH,* β*-carotene-linoleic acid, CUPRAC and ABTS^+^ assays. The metal chelating assay of extracts for Fe^2+^ was performed by using a spectrophotometer as the method reported previously^34^. The standard compound used was ethylenediaminetetraacetic acid (EDTA). The results of metal chelating assay were given as the % inhibition of 200 mg/mL concentration.

### Statistical analysis

The activities were performed three times each, and the mean ± standard error of the mean was recorded for the results. To determine significant differences between means, Student's test was utilized with a significance level of 0.05.

### Ethical approval

It is to confirm that all the methods described in the manuscripts are in accordance with the relevant guideline and regulations, as and when needed they are cited accordingly.

## Results

The analysis and comparison of polyphenol compounds using HPLC–DAD and study their biological activities were conducted on all three extracts obtained which include methanol, ultrasonic extraction and green extraction.

### Phenolic compositions

**Figure 1:**
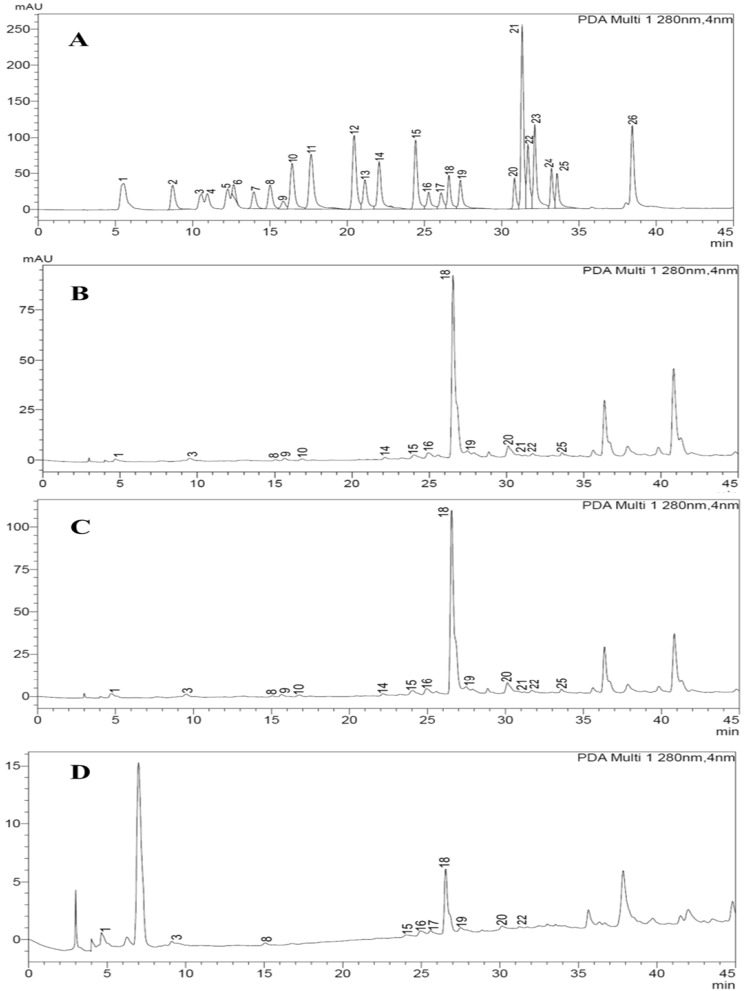
HPLC chromatograms of phenolic compounds, (**A**) standard phenolics (**B**) methanolic extract, (**C**) ultrasonic extract and (**D**) green extract.

**Figure 2:**
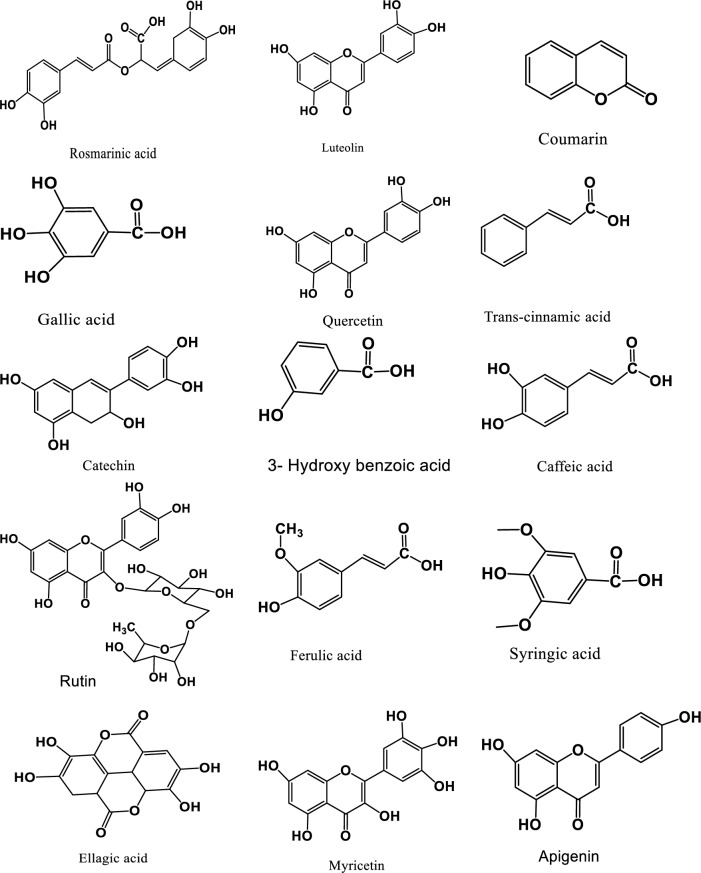
Structures of compounds identified in methanol ultrasonic and green extracts.

The phenolic composition of three types of extracts of *S. sclarea* including; methanolic extract, ultrasonic extract, and green extract, were determined and quantified in μg/g by using HPLC–DAD. Twenty-six reference phenolic compounds as standards were used. The results are reported in Table 1, showing that the Rosmarinic acid and Quercetin were in high quantity in all extracts, in ultrasonic extract both were quantified as 191.1 ± 0.27 μg/g and 25.01 ± 0.30 μg/g respectively, while in methanolic extract 179.5 ± 0.45 μg/g and 24.48 ± 0.22 μg/g respectively, and in green extract both phenolic were found as 90.31 ± 0.36 μg/g and 4.41 ± 0.25 μg/g, respectively. The phenolic compounds including; Gallic acid, Catechin, Caffeic acid, Syringic acid, Coumarin, Rutin, Rosmarinic acid, Myricetin, Quercetin, Luteolin and Apigenin were found in methanolic, ultrasonic and green extract of *S. sclarea* in variable quantities. While; 3-Hydroxy benzoic acid, Syringic acid, Ferulic acid, and Trans-cinnamic acid were only found in methanolic and ultrasonic extracts, whereas the Ellagic acid (3.40 ± 0.11 μg/g) was present only in green extract. The Protocatechuic acid, Pyrocatechol, Chlorogenic acid, P-hydroxy benzoic acid, and 6,7-Dihydroxy coumarin were not detected in any of the extract. The HPLC–DAD chromatograms of methanolic extract, ultrasonic extract, and green extract of *S. sclarea* are shown in Fig. 1. The structures of identified compounds in extracts were given in Fig. 2

**Table 1:** Phenolic composition of the methanol extract , ultrasonic extract and green extract of *S. sclarea* by HPLC–DAD (µg/g).

No.	Phenolic compounds	RT (min)	Methanol extract	Ultrasonic extract	(Citric acid:glycose)
1	Gallic acid	5.70	5.54 ± 0.10	5.23 ± 0.12	4.78 ± 0.15
2	Protocatechuic acid	8.75	–	–	–
3	Catechin	10.18	7.75 ± 0.21	7.42 ± 0.11	3.18 ± 0.10
4	Pyrocatechol	11.04	–	–	–
5	Chlorogenic acid	12.35	–	–	–
6	P-hydroxy benzoic acid	12.77	–	–	–
7	6,7-Dihydroxy coumarin	14.10	–	–	–
8	Caffeic acid	15.09	4.05 ± 0.08	4.41 ± 0.17	3.80 ± 0.16
9	3-Hydroxy benzoic acid	15.98	7.45 ± 0.33	7.81 ± 0.20	–
10	Syringic acid	16.56	3.31 ± 0.11	3.62 ± 0.24	–
11	Vanillin	17.78	–	–	–
12	P-Coumaric acid	20.56	–	–	–
13	Taxifolin	21.26	–	–	–
14	Ferulic acid	22.14	6.84 ± 0.18	5.21 ± 0.15	–
15	Coumarin	24.49	8.70 ± 0.10	8.77 ± 0.19	2.91 ± 0.10
16	Rutin	25.30	12.47 ± 0.25	16.21 ± 0.33	6.21 ± 0.28
17	Ellagic acid	26.11	-	-	3.40 ± 0.11
18	Rosmarinic acid	26.77	179.5 ± 0.45	191.1 ± 0.27	90.31 ± 0.36
19	Myricetin	27.35	8.27 ± 0.17	9.34 ± 0.21	3.75 ± 0.18
20	Quercetin	30.83	24.48 ± 0.22	25.01 ± 0.30	4.41 ± 0.25
21	Trans-cinnamic acid	31.33	5.20 ± 0.11	3.14 ± 0.19	-
22	Luteolin	31.70	9.15 ± 0.24	8.44 ± 0.16	2.15 ± 0.10
23	Hesperetin	32.14	–	–	–
24	Kaempferol	33.21	–	–	–
25	Apigenin	33.77	6.43 ± 0.16	5.83 ± 0.15	2.24 ± 0.15
26	Chrysin	38.40	–	–	–

### Biological activity

#### Minimum inhibitory concentration (MIC)

**Figure 3:**
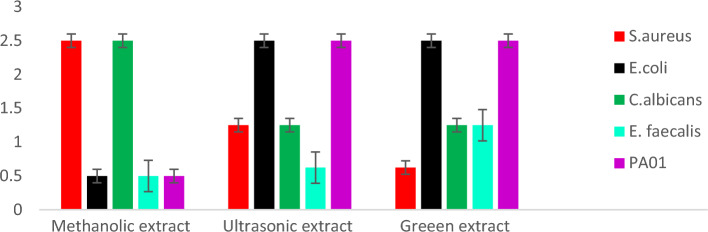
Antimicrobial activity *[(MIC)] values in mg/mL)]* of methanol, ultrasonic and green extracts of *S. sclarea.*

The minimum inhibitory concentration (MIC) of green extract compared to methanol and ultrasonic extracts of *S. sclarea* were evaluated against *E. coli*, *E. faecalis*, *C. albicans*, *S. aureus* and *P. aeruginosa* PA01 and the results are presented in Table 2 and Fig. 3. The methanolic extract of *S. sclarea* showed better activity with MIC value 0.5 mg/mL than ultrasonic and green extract on *E. coli* (with MIC value 2.5 mg/mL and 2.5 g/mL respectively), *E. faecalis* (with MIC value 0.625 mg/mL and 1.25 m/mL respectively) and *P. aeruginosa PA01* (with MIC value 2.5 g/mL), while ultrasonic extract and green extract showed similar activity on *C .albicans* and *P. aeruginosa PA01* with MIC value 2.5 mg/mL and 1.25 g/mL respectively. However, the green extract of *S. sclarea* showed an overall better inhibitory effect than the methanolic extract and ultrasonic extract against *S. aureus* strain with MIC value 0.625 mg/mL.

**Table 2:** Anti-microbial activity *[Minimum Inhibitory Concentration (MIC)] values in mg/mL)]* of methanol, ultrasonic and green extracts of *S. sclarea*.

Microorganisms	Methanol extract	Ultrasonic extract	Green extract
	MIC (mg/mL)		
*S. aureus*	2.5	1.25	0.625
*E.coli*	0.5	2.5	2.5
*C. albicans*	2.5	1.25	1.25
*E. faecalis*	0.5	0.625	1.25
*PA01*	0.5	2.5	2.5

**Figure 4:**
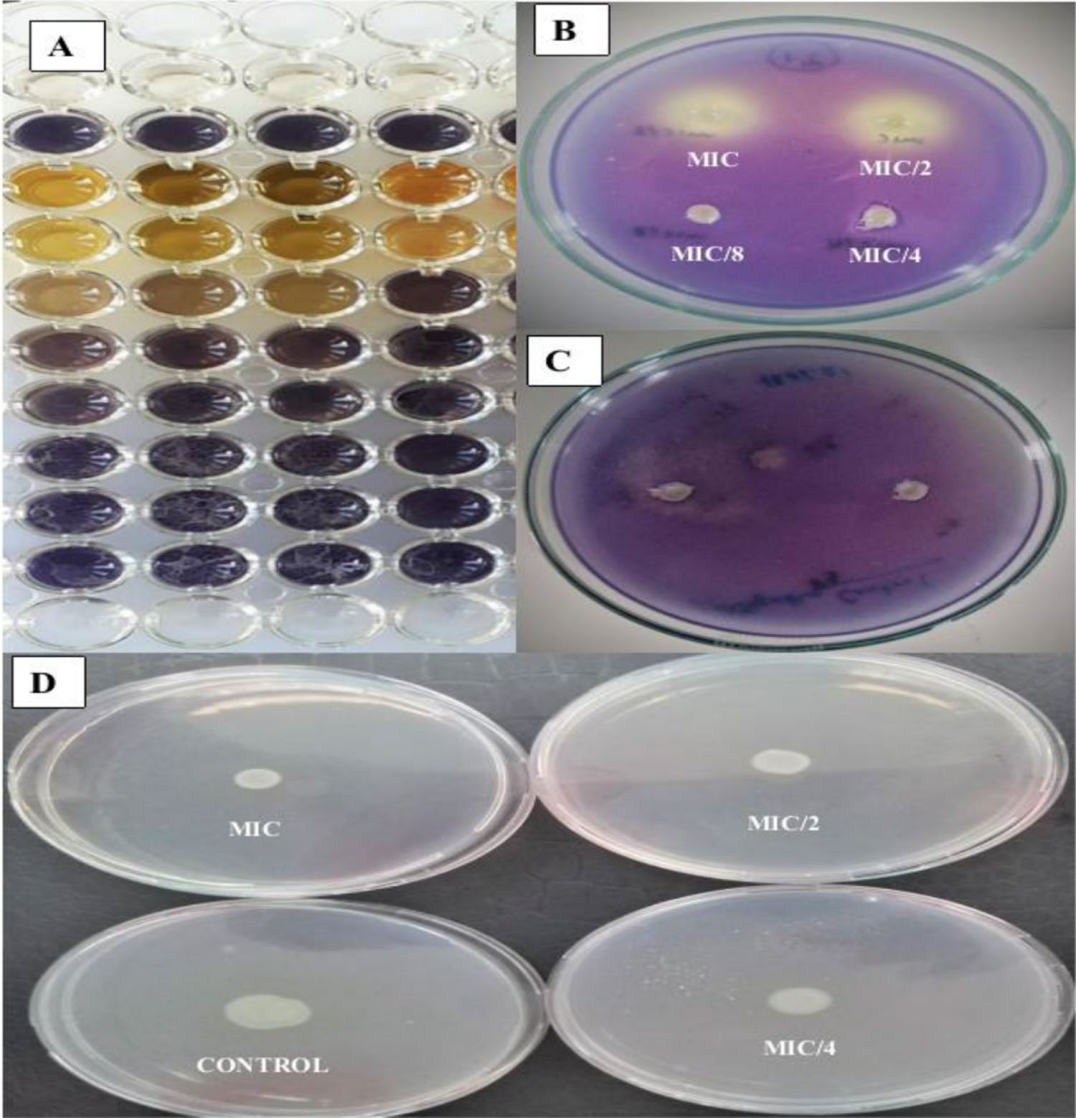
(**A**) Violacein inhibition plates against *C. violaceum* CV12472; (**B**)  quorum-sensing zinhibition plate against *C. violaceum* CV026; (**C**) quorum-sensing inhibition control plate; **D** = swarming motility inhibition plates against *P. aeruginosa* PA01.

Chromobacterium is a model organism used for measuring the quorum-sensing inhibition potential of bacteria. This is because it produces the bis-indole alkaloid pigment called violacein whose colour is easily measurable^35^. Using the *C. violaceum* CV12472, the concentration dependent decrease in violacein (purple) coloration reflects the inhibition of violacein which is a quorum sensing mediated process. The violacein plays the role of a signal molecule which aids bacteria cell-to-cell communication within colonies for coordinated behavior^36^. The extracts inhibited violacein inhibition and the percentage inhibition decreased with decreasing concentrations from MIC towards lower sub-MICs. This is reflected by the increasing violacein pigmentation down the plate as shown on Fig. 4. The mutant strain *C. violaceum* CV026 does not produce violacein except when an external acylhomoserine lactone is supplied to it. Chemical substances which can prevent this bacterium from producing the purple coloration in the presence of supplied acylhomoserine lactone hormone are known to possess anti-quorum sensing activity^37,38^. This is reflected by the brownish halos around the wells against the purple lawn as shown on Fig. 4, reflecting inhibition zones whose diameters are measured in millimeters.

#### Percentage biofilm inhibition

The biofilm inhibition of each extract against selected pathogens was evaluated at MIC and sub-MIC concentrations as described in Table 3. The results revealed that the methanolic extract of *S. sclarea* had better activity than ultrasonic and green extracts in inhibition of biofilm figuration of *C. albicans* and *E. coli*; whereat recorded inhibition on *C. albicans* 58.24 ± 1.02% and 24.68 ± 0.47% at MIC and MIC/2 concentration respectively. The biofilm inhibition of *E. coli* was recorded for methnolic extract as 46.14 ± 0.95 and 24.68 ± 0.47 at MIC and MIC/2 concentrations. The green extraction showed better biofilm inhibition than methanolic and ultrasonic extracts against strain of *S. aureus*, and *E. faecalis*; whereat recorded inhibition (47.26 ± 0.72% and 13.16 ± 0.24), (39.04 ± 1.24% and 15.85 ± 0.36) at MIC and MIC/2 consecutively.

**Table 3:** Anti**-**biofilm inhibition of methanol, ultrasonic and green extracts of *S. sclarea.*

Microorganisms	% Inhibition		
	Methanol extract	Ultrasonic extract	Green extract
*S. aureus*			
MIC	25.64 ± 0.41	37.42 ± 0.58	47.26 ± 0.72
MIC/2	8.67 ± 0.2	24.26 ± 0.36	13.16 ± 0.24
MIC/4	–	9.32 ± 0.11	–
MIC/8	–	–	–
*E. coli*			
MIC	46.14 ± 0.95	18.51 ± 0.32	36.75 ± 0.59
MIC/2	25.48 ± 0.38	9.39 ± 0.14	8.85 ± 0.1
MIC/4	–	–	–
MIC/8	–	–	–
*C. albicans*			
MIC	58.24 ± 1.02	–	–
MIC/2	24.68 ± 0.47	–	–
MIC/4	–	–	–
MIC/8	–	–	–
*E. faecalis*			
MIC	34.48 ± 1.06	–	39.04 ± 1.24
MIC/2	21.7 ± 0.65	–	15.85 ± 0.36
MIC/4	9.86 ± 0.45	–	–
MIC/8	–	–	–
PA01			
MIC	14.5 ± 0.42	11.5 ± 0.24	10.9 ± 0.01
MIC/2	–	–	–
MIC/4	–	–	–
MIC/8	–	–	–

#### Quorum sensing inhibition zones in *C. violaceum* CV026

Estimation of quorum sensing inhibition of methanolic, ultrasonic and green extracts of *S. sclarea* was carried out using *C. violaceum* CV026 strain and the results are reported in Table 4. The minimum inhibitory concentration (MIC) of the selected strain against each extract was measured prior, and the quorum sensing inhibition at MIC and sub-MIC concentrations were determined. The MIC values of methanolic, ultrasonic and green extracts of *S. sclarea* against *C. violaceum CV026* was recorded as 0.5 mg/mL, 0.25 mg/mL and 0.25 mg/mL respectively. Among the extracts tested, the ultrasonic extract showed a highest anti-quorum sensing activity with an inhibition zone of 15.5 ± 0.5 mm, 12.5 ± 0.2 mm, and 10.0 ± 0.1 mm at MIC, MIC/2 and MIC /4 concentration in turn, while the green extract showed slightly anti quorum sensing activity with an inhibition zone of 14.0 ± 1.1 mm, 12.0 ± 0.4 mm and 9.5 ± 0.5 mm at MIC, MIC/2 and MIC /4 concentrations in turn. The least anti-quorum sensing activity was recorded for methanolic extract with an inhibition zone of 13.5 ± 1.0 mm and 10.0 ± 0.5 mm at MIC and MIC/2 respectively; whereas no inhibition zone was recorded at MIC/4. None of the extracts showed any quorum sensing inhibition at a concentration of MIC/8.

**Table 4:** Quorum sensing inhibition zones in *C. violaceum* CV026 by test samples.

Sample code	Anti-quorum sensing inhibition zones (mm)				
	MIC (mg/mL)	MIC	MIC/2	MIC/4	MIC/8
Methanolic extract	0.5	13.5 ± 1.0	10.0 ± 0.5	–	–
Ultrasonic extract	0.25	15.5 ± 0.5	12.5 ± 0.2	10.0 ± 0.1	–
green extract	0.25	14.0 ± 1.1	12.0 ± 0.4	9.5 ± 0.5	–

**Figure 5:**
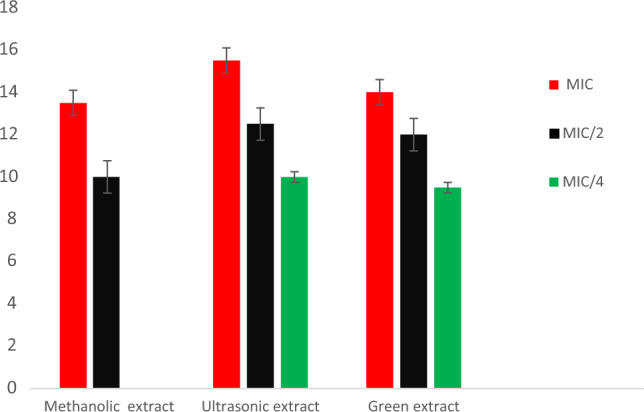
Quorum sensing inhibition zones in *C. violaceum* CV026 of *)]* of methanol, ultrasonic and green extracts of *S. sclarea.*

Consequently, quorum sensing inhibition potential were recorded as ultrasonic extract > green extract > methanolic extract as shown in Fig. 5.

#### Inhibition of violacein production

**Figure 6:**
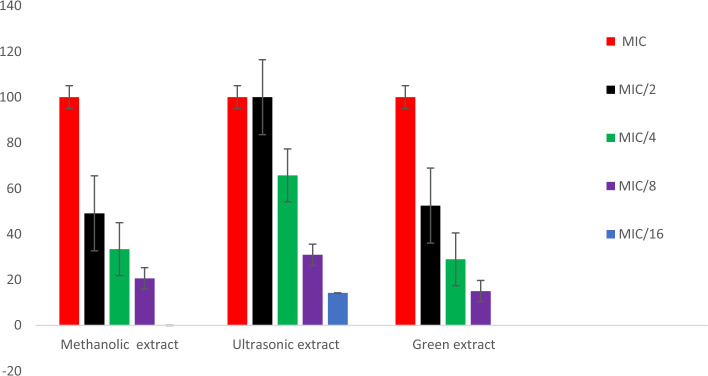
Violacein production inhibition in *C. violaceum* CV12472 of methanol, ultrasonic and green extracts of *S. sclarea.*

The green extract, methanolic extract and ultrasonic extract were tested for antimicrobial activity using *C. violaceum CV12472* to measure the inhibition of violacein production. The results reported in Table 5 and Fig. 6, all the extracts showed 100% violacein inhibition activity against *C. violaceum CV12472* at MIC values as 1.25 mg/mL, 0.625, 0.3125 mg/mL, respectively. The ultrasonic extract maintained 100% violacein inhibition even at MIC/2 concentration. Further, the percentage inhibition of violacein production by ultrasonic extract at sub-MIC concentration exhibited inhibition as 65.7 ± 1.5, 30.9 ± 1.8 and 05.5 ± 0.1 at MIC/4, MIC/8, MIC/16 and MIC/32. While, the percentage inhibition of violacein production by the green extract was 52.5 ± 2.5, 28.9 ± 1.2 and 15.0 ± 0.5 at MIC/2, MIC/4 and MIC/8, respectively. Likewise, the methanolic extract also showed percent inhibition of violacein production of 49.1 ± 0.9, 33.4 ± 1.1 and 20.6 ± 0.5 at a concentration of MIC/2, MIC/4, and MIC/8. Consecutively, hence the order of violacein production inhibition potential will be as ultrasonic extract > green extract > methanolic extract.

**Table 5:** Inhibition of violacein production in *C. violaceum* CV12472 by test samples.

Sample	MIC (mg/mL)	Violacein inhibition (%)					
		MIC	MIC/2	MIC/4	MIC/8	MIC/16	MIC/32
Methanolic extract	0.625	100 ± 0.00	49.1 ± 0.9	33.4 ± 1.1	20.6 ± 0.5	–	–
Ultrasonic extract	0.3125	100 ± 0.00	100 ± 0.00	65.7 ± 1.5	30.9 ± 1.8	14.2 ± 0.4	05.5 ± 0.1
Green extract	1.25	100 ± 0.00	52.5 ± 2.5	28.9 ± 1.2	15.0 ± 0.5	–	–

#### Swarming motility inhibition

**Figure 7:**
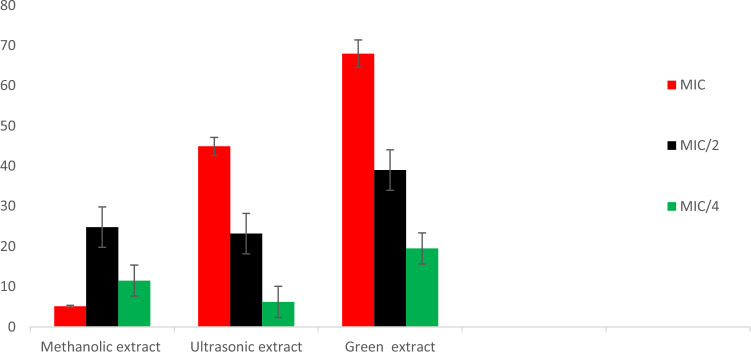
Swarming motility inhibition on *P. aeruginosa* PA01 of methanol, ultrasonic and green extracts of *S. sclarea.*

The methanolic, ultrasonic and green extracts of *S. sclarea* were tested for swarming motility inhibition against the *P. aeruginosa* PA01 at MIC, MIC/2, and MIC/4 concentrations, as displayed in Table 6. The green extraction showed significant swarming motility inhibition compared to methanolic and ultrasonic extracts under similar conditions. The inhibition of swarming motility by green extraction was 68.0 ± 2.1%, 39.6 ± 0.8% and 19.5 ± 0.6% at MIC, MIC/2 and MIC/4 concentrations, respectively. While; the inhibition of swarming motility by the methanolic extract was 51.3 ± 1.8%, 24.8 ± 0.9% and 11.5 ± 0.4% at MIC, MIC/2 and MIC/4 concentrations, respectively. However, the inhibition of swarming motility by the ultrasonic extract was 44.9 ± 0.5%, 23.2 ± 1.2% and 06.2 ± 0.1% at concentrations of MIC, MIC/2 and MIC/4, respectively. Consequently, the order of the inhibition efficacy was as green extracts > methanolic extract > ultrasonic extract, as shown in Fig. 7.

**Table 6:** Swarming motility inhibition on *P. aeruginosa* PA01 by test samples.

Sample code	Swarming inhibition (%)		
	MIC	MIC/2	MIC/4
Methanolic extract	51.3 ± 1.8	24.8 ± 0.9	11.5 ± 0.4
Ultrasonic extract	44.9 ± 0.5	23.2 ± 1.2	6.2 ± 0.1
Green extract	68.0 ± 2.1	39.6 ± 0.8	19.5 ± 0.6

### Enzyme inhibition activities

#### Anticholinesterase activity

**Figure 8:**
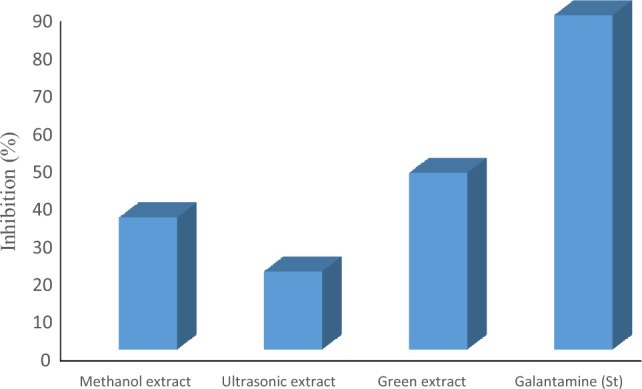
AChE inhibition of methanolic extract, ultrasonic extract, and green extract of *S. sclarea*.

**Figure 9:**
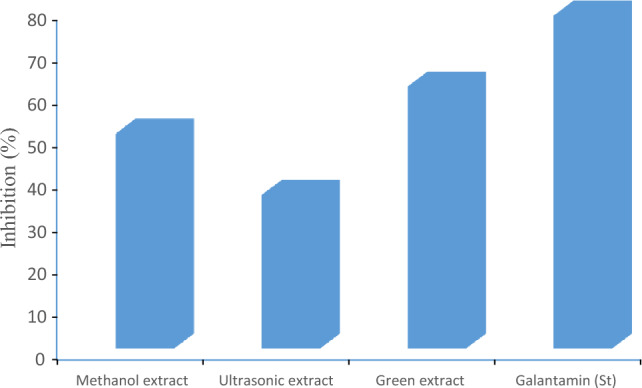
BCHE inhibition of methanolic extract, ultrasonic extract, and green extract of *S. sclare.*

The ability of the methanolic, ultrasonic, and green extracts of *S. sclarea* to inhibit certain selected enzymes, including acetylcholinesterase (AChE) and butyrylcholinesterase (BChE) were evaluated in vitro and the obtained results are displayed in Table 7. Very moderate percentage inhibition by each tested extract was recorded against AChE. Maximum AChE inhibition 47.00 ± 1.50% was shown by green extract, followed by methanolic extract (35.20 ± 0.57%) and ultrasonic extract (20.97 ± 0.13%). All the extracts had IC_50_ values over 200 μg/mL for AChE inhibition. Among the extracts tested, the green extract showed maximum 61.79 ± 0.63% inhibition against BChE with 131.6 ± 0.98 µg/mL IC_50_ value. The methanolic extract inhibited 50.70 ± 0.94% BChE with IC_50_ 192.4 ± 1.25 µg/mL, while ultrasonic extract recorded weak inhibition (36.21 ± 0.50% at > 200 µg/mL) against BChE. Consequently, potential inhibition of AChE and BChE could be in order as green extracts > methanolic extract > ultrasonic extract, Figs. 8 and 9.

**Table 7:** Cholinesterase and Urease inhibitory activities of the  tested extracts.

Extracts	AChE		BChE		Urease inhibitory	
	Inhibition (%) (at 200 µg/mL)	IC_50_ (µg/mL)	Inhibition (%) (at 200 µg/mL)	IC_50_ (µg/mL)	Inhibition (%) (at 200 µg/mL)	IC_50_ (µg/mL)
Methanol extract	35.20 ± 0.57	> 200	50.70 ± 0.94	192.4 ± 1.25	52.31 ± 0.74	187.5 ± 1.32
Ultrasonic extract	20.97 ± 0.13	> 200	36.21 ± 0.50	> 200	55.12 ± 0.88	171.6 ± 0.95
Green extract	47.00 ± 1.50	> 200	61.79 ± 0.63	131.6 ± 0.98	11.46 ± 0.82	> 200
Galantamine	88.70 ± 0.45	5.50 ± 0.18	78.50 ± 0.25	40.05 ± 0.30	*NT*	*NT*
Thiourea	*NT*	*NT*	*NT*	*NT*	85.75 ± 0.40	8.15 ± 0.33

Values represent the means ± SEM of three parallel sample measurements (*p* < 0.05).

*NT=* Not tested.

#### Anti-urease activity

**Figure 10:**
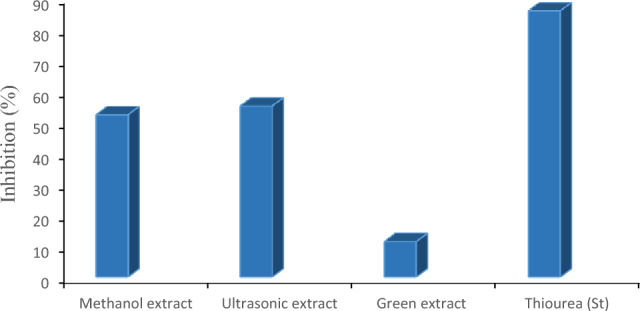
Urease enzyme inhibition of methanolic extract, ultrasonic extract, and green extract of *S. sclarea.*

Table 7 presents the ability of methanolic, ultrasonic and green extracts of *S. sclarea* to inhibit the urease enzyme. The results clearly show that the ultrasonic extract and methanolic extract have moderated inhibition potential (55.12 ± 0.88% and 52.31 ± 0.74%) with IC_50_ 171.6 ± 0.95 µg/mL and 187.5 ± 1.32 µg/mL respectively, while the green extract showed merely 11.46 ± 0.82% urease inhibition activity, Fig. 10.

### Antioxidant activities

#### β-Carotene-linoleic acid assay

The inhibition of lipid peroxidation activity of methanol extract, green extract and ultrasonic extracts of *S. sclarea* were evaluated, and the results are presented in Table 8. The green and methanolic extracts exhibited potent activity with IC_50_ (5.61 ± 0.47 µg/mL) and (5.37 ± 0.27 µg/mL) respectively; while ultrasonic extract showed appreciable activity with IC_50_ value 36.58 ± 0.63 µg/mL. The activity of methanolic extract and the green extract was relatively close to each other and to the inhibition exhibited by standard BHA (IC_50_ = 1.46 ± 0.03 μg/mL), and α-Tocopherol (IC_50_ = 2.25 ± 0.04 μg/mL).

**Table 8:** Antioxidant activity of the extracts of *S. sclarea* by the β-carotene-linoleic acid, DPPH^**·**^, ABTS^**·**+^, CUPRAC, and metal-chelating assays^a^.

Antioxidant activity					
Extracts	β-Carotene-linoleic acid assay IC_50_ (µg/mL)^a^	DPPH^**·**^ assay IC_50_ (µg/mL)	ABTS^**·**+^ assay IC_50_ (µg/mL)	CUPRAC assay A_0.5_ (µg/mL)^b^	Metal chelating assay IC_50_ (µg/mL)
Methanol extract	5.37 ± 0.27	16.31 ± 0.23	6.50 ± 0.45	12.28 ± 0.12	47.22 ± 0.54
Ultrasonic extract	36.58 ± 0.63	> 100	52.25 ± 0.73	76.48 ± 0.27	80.91 ± 1.20
Green extract	5.61 ± 0.47	19.20 ± 0.70	8.64 ± 0.63	17.22 ± 0.36	42.35 ± 1.04
BHA^c^	1.46 ± 0.03	19.70 ± 0.25	12.85 ± 0.52	25.12 ± 0.01	*NT*
α-Tocopherol^c^	2.25 ± 0.04	38.70 ± 0.32	34.50 ± 0.48	85.36 ± 0.02	*NT*
EDTA^c^	*NT*	*NT*	*NT*	*NT*	5.51 ± 0.53

*NT* not tested.

^a^IC_50_ values represent the means ± SEM of three parallel measurements (*p* < 0.05).

^b^A_0.50_ values represent the means ± SEM of three parallel measurements (*p* < 0.05).

^c^Reference compounds.

#### DPPH^***·***^ assay

The free radical scavenging activity of each methanolic, ultrasonic and green extract(s) of *S. sclarea* is presented in Table 8. The methanol extract and green extract exhibited considerable free radical scavenging activity with IC_50_ = 6.31 ± 0.23 μg/mL and IC_50_ = 19.20 ± 0.70 μg/mL, respectively. Both these extracts showed better activity than the standards BHA (IC_50_ = 19.70 ± 0.25 µg/mL) and α-tocopherol (IC_50_ = 38.70 ± 0.32 µg/mL). No activity was noted by the ultrasonic extract.

#### ABTS^***·***+^ assay

The results of antioxidant activity by ABTS^**·+**^ assay of methanolic extract, green extract and ultrasonic extract of *S. sclarea* is presented in Table 8*.* The highest ABTS^**·+**^ antioxidant activity was shown by the methanolic extract with IC_50_ = 6.50 ± 0.45 µg/mL followed by green extract (IC_50_ = 8.64 ± 0.63 µg/mL), whereas the ultrasonic extract showed moderate antioxidant activity with IC_50_ = 52.25 ± 0.73 µg/mL. The considerable activity exhibited by green and methanolic extracts were even better than that of standards drugs BHA (IC_50_ = 12.85 ± 0.52 µg/mL) and α-Tocopherol (IC_50_ = 34.50 ± 0.48 µg/mL).

#### CUPRAC assay

The results of CUPRAC assay of methanolic extract, green extract and ultrasonic extract of *S. sclarea* are reported in Table 8, methanolic extract and green extract exhibited significant activity with (A_0.5_ = 12.28 ± 0.12 µg/mL) and (A_0.5_ = 17.22 ± 0.36 µg/mL) respectively; while ultrasonic extract showed moderate activity with A_0.5_ = 76.48 ± 0.27 µg/mL, the methanolic and green extracts highly potent and showed even better activity than standers BHA (A_0.5_ = 25.12 ± 0.01 µg/mL) and α-Tocopherol (A_0.5_ = 85.36 ± 0.02 µg/mL).

#### Metal chelating assay

**Figure 11:**
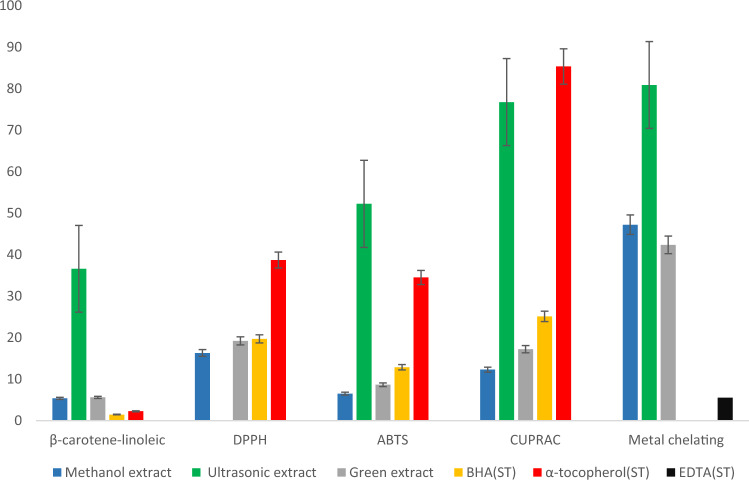
Antioxidant activity (IC50 μg/mL) of methanolic extract, ultrasonic extract, and green extract of *S. sclarea* evaluated by different methods.

The green extract, methanol extract and ultrasonic extract of *S. sclarea* were tested for antioxidant activity by using metal chelating assay and the results are presented in Table 8. Among of the tested extracts, the green extract exhibited highest metal chelating activity with IC_50_ = 42.35 ± 1.04 µg/mL, followed by methanolic extract with IC_50_ = 47.22 ± 0.54 µg/mL while the ultrasonic extract showed minimal antioxidant activity with IC_50_ = 80.91 ± 1.20 µg/mL. For comparison the antioxidant activities of all extract(s) as tested by different methods is depicted in Fig. 11.

## Discussion

This study was designed to analyze the presence of various phenolic compounds of *S. sclarea* by extraction through the green extraction process and its comparison with traditional methanol extraction and ultrasonic extraction. The phenolic compounds were abundant in all the tested extracts with variation in their quantities. In the methanolic and ultrasonic extracts fourteen phenolic compounds including 3-Hydroxy benzoic acid, Syringic acid and Trans-cinnamic acid were exclusively available were not present in green extract. Likewise, in the green extract, 12 compounds were detected one of them was Ellagic acid, that was not found in other extracts. Phenolic compounds are secondary metabolites that contain benzene rings, with one or more hydroxyl groups, and range from simple phenolic molecules to highly polymerized compounds^39^. The phenolic compound plays an important role as an antioxidant agent, providing protection to the cells of organisms against oxidative damage^40^. Furthermore, phenolic compounds have the efficacy of different therapeutic properties such as anti-aging, anti-inflammatory, anti-diabetic and anti-proliferative agents^41^.

The results of antibacterial activity against the selected bacteria strains, including *S. aureus, E. coli, C. albicans, E. faecalis,* and *P. aeruginosa* PA01 showed appreciable activity of methanolic, ultrasonic and green extracts of *S. sclarea.*

This forced us to evaluate further the potential inhibition of selected extracts toward bacterial biofilm formation. The biofilms are microbial communities, in which bacterial cells are embedded in a self-generated matrix of lipids, exopolysaccharides (EPS), proteins, and nucleic acids. This matrix blocks the entry of antimicrobial agents into cells^42,43^, hence providing the resistance to many bacteria against numerous drugs^44^. The results of this study proved that the methanolic, ultrasonic and green extracts had inhibited the biofilm figuration, where methanolic extract showed inhibition against figuration of biofilm of four types of microbial strains including *S. aureus*, *E. coli*, *C. albicans* and *E. faecali*, similarly, the green extract showed good inhibition for figuration biofilm of four of bacteria strains that were *S. aureus*, *E. coli*, *E. faecalis* and *P. aeruginosa PA01*, while ultrasonic extract exhibited affection against biofilm formation of three of bacteria strains were *S. aureus*, *E.coli* and *P. aeruginosa PA01*. The observed results may be attributed to the destabilization of the cell membrane which could be caused by phenolic compounds present at concentrations below the MIC^45^. Additionally, the studies reported that formation of biofilm depends on several factors, including quorum sensing signaling^46^. However, detection of quorum inhibition is likely played a critical role in blocking biofilm formation. Further, studies are needed to assess the mechanisms of biofilm inhibition observed in this study and many others. Many researchers have also reported the inhibition of anti-biofilm formation. The methanol extract of *Capparis spinosa* (capers) inhibited the formation of biofilm biomass in *P. mirabilis*, *E. coli*, and *P. aeruginosa PAO1*^47^.

Quorum sensing indicates to the connection system of bacteria at the molecular level where the bacteria react to different environmental signals and perform assignments as a group such as; virulence gene expression, biofilm formation, migration to more favorable environments, bacteriocin, antibiotic production and pigment production^48,49^.

A disarrangement in any of the steps needed for the quorum sensing communication could lead to overlap with microbial pathogenesis and help in bacterial control^50^. In the current study, all tested extracts showed inhibition of quorum sensing, the order of ability inhibition was as ultrasonic extract > green extract > methanolic extract. The Phenolic compounds were reported previously as anti-quorum sensing agent^51^, furthermore, numerous investigations demonstrated the capacity of phenolic compounds as an excellent inhibitor of violacein production. The studies found Caffeic acid reduced violazine production by up to 75%, Gallic acid by up to 59%, Oleuropine glycoside to 51% and Epicatechin 33%, 51%. Ferric acid by up to 72%, Floridine up to 48%^52,53^. In the current investigation, all extracts exhibited significant violacein production inhibition and the ultrasonic extract showed the highest inhibition, on the other hand, the phenolic compounds including Caffeic acid and Gallic acid identified in all extracts tested, therefore we hypothesize that the inhibition of violacein production observed in this investigation related to the presence of phenolic compounds acting individually or together in synergistically effect. Swarm motility is a quick and harmonious, translocation of a bacterial population to solid or semi-solid surfaces and is an example of bacterial multicellularity and swarm behaviour^54^. Bacterial swimming motility affects many pathogen–host interactions, and several pathogens are also capable of migrating multicellular swarms. The bacterial motility of swimming may be related to biofilm formation^55^. The phenolic compounds documented earlier as swarming motility inhibitors, for instance, the study by Borges et al. had exhibited inhibition capability of phenolic compounds including Gallic acid, Caffeic acid and  Ferulic acid against swarming motility of *P. aeroginosas*, *S. aureus*, *E. coli*, and *L. monocytogenes*^52,56^, their results proved the significant potential of these phenolic compounds as swarming motility inhibitors agent. In the current study, as mentioned above the gallic acid, caffeic acid and ferulic acid were detected in all extracts tested, therefore, the potential inhibition swarming motility that have shown by extracts in our results may be linked to the existence of the phenolic compounds in the extracts.

The ability of green extract, ultrasonic extract and metanolic extract of *S. sclarea* to inhibit AChE, BChE and Urease enzymes were evaluated in this study for the first time. Our results proved the green extract showed as choline esterase (both AChE and BChE) inhibition better than conventional extracts including methanol and ultrasonic extract. Several studies have documented that the natural products and their derivatives can remedy Alzheimer's disease by inhibiting acetylcholinesterase enzyme (AChE) and butyrylcholinesterase enzyme (BChE)^57,58^. The essential oil extracted from *Salvia lavandulaefolia* and *O. syriacum* have shown significant anticholinesterase inhibitory activity^59,60^. Moreover the phenolic compounds obtained from Salvi species were found as considerable (AChE) and (BChE) inhibitory^61^, furthermore the phenolic compounds have previously described as AChE and BChE inhibitory molecules^62,63^. The inhibition of (AChE) and (BChE) observed in our investigation could be attributed to the presence of phenolic compounds detected already in extracts. Meanwhile, the results obtained from the urease inhibition assay showed the methanolic and ultrasonic extracts possess moderate urease inhibition however the green extract showed minimal inhibition against urease the enzyme. The earlier studies have reported that phenolic compounds such as Gallic acid and Quercetin possess a great capacity to inhibit urease enzyme^64,65^, and although the compounds mentioned were present in extracts tested, however, the green extract showed little inhibition which can be attributed to the state of the extract, the other compounds in an extract could interfere with each other and the activity of a known compound may be masked by another in the mixture.

Antioxidant agents play a vital part in food preservation via inhibiting oxidation processes and contributing to health promotion made by many food supplements, nutrients and functional food ingredients^66^. In the current investigation, antioxidant activity was evaluated by different methods including *β*-carotene-linoleic acid assay, DPPH^**·**^ assay, ABTS^**·**+^ assay, CUPRAC assay and metal chelating assay. The free radical quenching abilities of extracts including methanolic, ultrasonic and green extracts of *S. sclarea* were performed by using DPPH and ABTS assays. DPPH is a stable free radical, it can be easily squelched, by an antioxidant agent which it loses this absorption by accepting an electron or a radical species^67^. In DPPH assay; we found the green extract and methanolic extract showed considerable potential in scavenging free radicals even higher than the standards used (BHA and α-tocopherol). Likewise, the ABTS tests used to determine hydrogen donation and chain-breaking capacity of the tested sample, we found that the methanolic and green extracts showed competitive ABTS radical scavenging capacity significantly greater than the references used. Meanwhile, The antioxidant capacity was also estimated using the carotene/linoleic acid bleach test, the activity test for neutralizing free linoleate radicals as well as free radicals that are shaped in the system and attack highly unsaturated *β*-carotene patterns^68^. As the results we mentioned above the green extract and methanolic extract had almost the same activity, whereat both extracts exhibited significant activity. The activity observed might be attributed to the presence of antioxidant agents in extracts such as phenolic compounds that could prevent the spread of beta carotene demolition by linoleate or any other radicals that may be shaped within the system.

Cu^2+^ reduction is used for the determination of electron donation activity. The ability of methanolic, ultrasonic, and green extracts to reduce Cu^2+^ to Cu^1+^ were tested. All tested extracts showed activity to reduce Cu^2+^, however, the methanol and green extracts showed significant ability in reducing Cu^2+^ to Cu^1+^ even more than standers used, the ability to reduce Cu^2+^ to Cu^1+^ of extracts tested was in the order of methanolic extract > green extract > ultrasonic extract.

Frequently, the capacity of metal chelation is assessed by determining the chelating effect of antioxidants to ferrous ion. In the current investigation the capacity of ferrous ion chelating of methanolic, green extract, and ultrasonic extracts of *S. sclarea* was evaluated via assessing Fe(II) ferrozine test system, where the results indicated as EDTA equivalents. It is already mentioned in the results section that the green extract exhibited the highest activity among the extracts tested, followed by methanolic extract and ultrasonic extract. The results are in close agreement with the literature reported for Salvia species such as; *Salvia virgata* Jacq^69^, *Salvia officinalis* L.^70^, Salvia fruticosa^71^, and *Salvia verticillata*^72^ to possess antioxidant properties substantially.

The antioxidant activity observed in this investigation could be linked to the existence of phenolic compounds abundantly which are demonstrated as antioxidant agents such as Quercetin^73^, Rosmarinic acid^74^ and Gallic acid^75^.

## Conclusion

Based on the results reported above the phenolic profile identified by HPLC–DAD revealed Rosmarinic acid was the major phenolic compound in all extracts. Ellagic acid was found only in the green extract, while 3-Hydroxy benzoic acid and Syringic acid, Ferulic acid, and Trans-cinnamic acid were detected in methanolic and ultrasonic extracts, while were absent in green extract. The green extract had better anti-biofilm activity than methanolic and ultrasonic extracts on *S. aureus* and *E. faecalis*; while methanolic extract had anti-biofilm activity than green and ultrasonic extracts on *E. coli, C. albicans* and *P. aeruginosa PA01* at the same concentration, all extracts showed significant inhibition violacein production inhibition on *C. violaceum* CV12472, among the extracts tested, the ultrasonic extract showed as strongest inhibition of violacein production on *C. violaceum* CV12472, followed by green extract and methanolic extract, respectively. The green extract showed the highest swarming motility inhibition on *P. aeruginosa PA01* better than methanolic extract and ultrasonic extract. The green extract exhibited good activity against BChE and AChE better than the methanolic and ultrasonic extracts, while ultrasonic extract and methanolic extract showed activity against urease enzyme, respectively better than the green extract. Furthermore, green and methanolic extracts showed considerable activity for *β*-carotene-linoleic acid, DPPH^**·**^, ABTS^**·**+^ and CUPRAC assay better than ultrasonic extract. However, green extract showed activity better than the methanolic and ultrasonic extracts in metal chelating assay. To the best of our knowledge the current investigation is the first paper demonstrated the therapeutical properties of *S. sclarea* by using green extraction. Moreover, the current study confirmed the efficiency of the green solvent in the extraction of bioactive compounds from natural sources as the new promising solvent, nontoxic cheaper, and friendly to the environment.

## Data Availability

All data generated or analysed during this study are included in this published article.
